# Parameters of biliary hydrodynamic injection during endoscopic retrograde cholangio-pancreatography in pigs for applications in gene delivery

**DOI:** 10.1371/journal.pone.0249931

**Published:** 2021-04-28

**Authors:** Yuting Huang, Robert L. Kruse, Hui Ding, Mohamad I. Itani, Jonathan Morrison, Zack Z. Wang, Florin M. Selaru, Vivek Kumbhari

**Affiliations:** 1 Division of Gastroenterology & Hepatology, Department of Medicine, The Johns Hopkins University School of Medicine, Baltimore, Maryland, United States of America; 2 Department of Medicine, University of Maryland Medical Center Midtown Campus, Baltimore, Maryland, United States of America; 3 Department of Pathology, The Johns Hopkins University School of Medicine, Baltimore, Maryland, United States of America; 4 Division of Gastroenterology and Hepatology, Renji Hospital, Shanghai Jiao Tong University, Shanghai, China; 5 R Adams Cowley Shock Trauma Center, University of Maryland School of Medicine, Baltimore, Maryland, United States of America; 6 Division of Hematology, Department of Medicine, The Johns Hopkins University School of Medicine, Baltimore, Maryland, United States of America; 7 Division of Gastroenterology & Hepatology, Department of Medicine, Mayo Clinic College of Medicine and Science, Jacksonville, Florida, United States of America; Texas A&M University, UNITED STATES

## Abstract

The biliary system is routinely accessed for clinical purposes via endoscopic retrograde cholangiopancreatography (ERCP). We previously pioneered ERCP-mediated hydrodynamic injection in large animal models as an innovative gene delivery approach for monogenic liver diseases. However, the procedure poses potential safety concerns related mainly to liver or biliary tree injury. Here, we sought to further define biliary hydrodynamic injection parameters that are well-tolerated in a human-sized animal model. ERCP was performed in pigs, and hydrodynamic injection carried out using a novel protocol to reduce duct wall stress. Each pig was subjected to multiple repeated injections to expedite testing and judge tolerability. Different injection parameters (volume, flow rate) and injection port diameters were tested. Vital signs were monitored throughout the procedure, and liver enzyme panels were collected pre- and post-procedure. Pigs tolerated repeated biliary hydrodynamic injections with only occasional, mild, isolated elevation in aspartate aminotransferase (AST), which returned to normal levels within one day post-injection. All other liver tests remained unchanged. No upper limit of volume tolerance was reached, which suggests the biliary tree can readily transmit fluid into the vascular space. Flow rates up to 10 mL/sec were also tolerated with minimal disturbance to vital signs and no anatomic rupture of bile ducts. Measured intrabiliary pressure was up to 150 mmHg, and fluid-filled vesicles were induced in liver histology at high flow rates, mimicking the changes in histology observed in mouse liver after hydrodynamic tail vein injection. Overall, our investigations in a human-sized pig liver using standard clinical equipment suggest that ERCP-guided hydrodynamic injection will be safely tolerated in patients. Future investigations will interrogate if higher flow rates and pressure mediate higher DNA delivery efficiencies.

## Introduction

Hydrodynamic injection represents a promising method of non-viral gene therapy. The technique consists of briefly exposing tissues to increased pressure and/or fluid volume levels, transiently causing disruptions in cell membranes, thereby mediating delivery of DNA inside the cell [1]. Hydrodynamic injection was first pioneered for liver gene delivery, but has subsequently been shown to also mediate delivery in the skin, muscle, and kidney [2–4]. Beyond the delivery of DNA inside the cell, hydrodynamic injection has also been shown to mediate delivery of siRNA and proteins inside hepatocytes [5].

Hydrodynamic tail vein injection (HTVI) into mouse liver involves administration of 10% body fluid volume DNA solution in 4–7 seconds, yielding expression of delivered DNA throughout the liver [6, 7]. Of note, modulating volume and/or flow rate led to significant differences in transfection efficiency [7]. Hydrodynamic injection in mice causes fluid overload and temporary right heart failure, resulting in hepatic congestion of fluid [8]. While the technique may cause temporary liver damage in a portion of hepatocytes, histology and liver enzymes normalize within several days [9, 10]. Delivered DNA appears to be expressed into protein as early as 10 minutes after hydrodynamic injection, suggesting direct nuclear delivery of at least a fraction of DNA [11, 12]. Large, fluid-filled vesicles also transiently form post hydrodynamic injection, which may also help deliver DNA into the nucleus [13, 14]. Once inside the liver cells, DNA is stable due to slowly dividing hepatocytes, although it can be slowly epigenetically silenced over time [15, 16].

While hydrodynamic injection is a common method for liver gene therapy in mice, the technique is challenging in large animals, that cannot otherwise tolerate large vascular disturbances [1]. In an attempt to overcome this, a liver lobe/segment approach has been taken, whereby a closed vascular system is created via catheterization and occlusion balloons [17]. Hydrodynamic injection into this system creates a local increase in fluid pressure and local gene delivery, sparing systemic cardiovascular effects. Through this method, hydrodynamic gene delivery into the liver of pigs and dogs has been demonstrated [9]. Proving applicability to human liver, proof of concept experiments for hydrodynamic injection observed gene expression in *ex vivo* human liver segments [18]. Unfortunately, the first clinical trial using hydrodynamic injection of selective liver segments with thrombopoietin-expressing DNA in 14 cirrhotic patients with thrombocytopenia did not demonstrate clear expression [19].

Despite these advances, gene expression efficiency of vascular hydrodynamic approaches in large animals is markedly lower than mouse hydrodynamic injection and requires large quantities of fluid, DNA, as well as rapid injection velocity [18]. Moreover, given the complexity of selective vascular catheterization, translation to patients is cumbersome. The procedures would be invasive, posing potential complications such as tissue ischemia, thrombosis, and bloodstream infection. Thus, the gene therapy community has largely continued to focus on viral vector approaches.

The biliary system offers an alternative to vascular-mediated hydrodynamic injection for gene delivery into the liver. Non-viral gene therapy through the biliary tract was first demonstrated in dogs [20], with subsequent studies showing gene delivery in rat models [21–23]. In these studies, the biliary system was accessed through surgical means with a needle placed in the common hepatic duct (CHD) and surgical tie preventing antegrade biliary flow. Biliary gene transfection had comparable delivery efficiency to vascular systems [20]. The first study in a large animal to demonstrate a minimally invasive methodology to deliver genes by hydrodynamic injection in the biliary tree was published by our group [24]. We showed the feasibility of using endoscopic retrograde cholangio-pancreatography (ERCP) to deliver genes via hydrodynamic injection into the livers of pigs [24]. Gene expression was found in all five pig liver lobes after hydrodynamic injection with a power injector, commonly used to inject radiocontrast for blood vessel visualization [25].

Due to the potential trauma to liver tissue and bile ducts, we further investigated the safety and limitations of ERCP-mediated hydrodynamic injection, which is crucial for translation into humans. Herein, we explore a series of hydrodynamic injection parameters within pigs by ERCP, modeling potential clinical scenarios in patients. We analyzed the toxicity in the pigs as a function of a range of injection parameters, determined tolerance to multiple injections, and examined the mechanism of hydrodynamic injection through studying liver histology in comparison to mouse liver hydrodynamic injection.

## Materials and methods

### Animal experiments

All animal experiments were conducted under the approval of the institutional animal care and use committee (IACUC) of Johns Hopkins Hospital (protocol #SW19M428) and University of Maryland School of Medicine (protocol #0720003) and adhere to the guidelines of the NIH Guide for the Care and Use of Laboratory Animals.

Yorkshire pigs (*Sus scrofa domestica*) were acquired, weighing 35–54 kg. Pigs (8 total) were provided by Archer Farms (Darlington, Maryland). Pigs were housed in cages either singly or in pairs with different toys for enrichment, water ad libitum, and food provided each day. A detailed protocol of the biliary hydrodynamic injection procedure was previously described [24]. Briefly, after pigs were anesthetized and placed supine, a duodenoscope was inserted and positioned such that the biliary orifice in the duodenal bulb was *en face*. Under fluoroscopic guidance (Phillips Allura C-arm), the bile duct was cannulated with a triple lumen sphincterotome and hydrophilic guidewire. A cholangiogram was attained after injection of 5–10 mL of radio opaque contrast (Omnipaque, 350 mg/mL; GE Health Co). The sphincterotome was exchanged for a stone extraction balloon which was inflated to 12mm in the common hepatic duct.

Hydrodynamic injections were performed using a power injector (MEDRAD® Mark 7 Arterion) that contains up to 150 mL and can inject up to 50 mL/sec at a maximum of 1200 pounds per square inch (psi). For each injection, 25% contrast solution diluted with 0.9% saline solution was used to allow for real-time visualization to evaluate hepatic distribution and acinarization. In one pig, 5 milligrams of plasmid DNA, pCLucf, isolated with a gigaprep kit (Zymo Research) and dissolved into 0.9% saline solution and subsequently injected. pCLucf was a gift from John Schiller (Addgene plasmid # 37328). For the acute pig studies, several different injection parameters were tested as described in **Table 1**. For the day 1 studies, parameters of 4 mL/sec at 40 mL volume were utilized in pigs, while day 14 studies used 2 mL/sec at 30 mL volume in pigs. Between each injection, at least five minutes were allowed to lapse in time, and contrast was verified to be no longer visualized on fluoroscopy prior to repeat injection. For several experiments, a pressure catheter (FOP-M260, FISO Technologies) was advanced through the guidewire channel with the sensor positioned 1 cm beyond the distal tip of the catheter, allowing it to measure intrabiliary pressures. Pressure readings were monitored in real-time by the connection of the catheter to a computer able to illustrate pressure tracings in real time. At the completion of the study, pigs were euthanized using potassium chloride overdose (>2mmol/kg) following by verification of cardiac arrest.

**Table 1:** Biliary hydrodynamic injection parameters used in the acute pig studies.

Pig #1					
Injection Attempt	Volume (mL)	Flow rate (mL/sec)	Pressure (psi)	Port	Notes
1	30	2	999	Injection (small)	Well-tolerated by pig, no power injector deviation
2	30	4	999	Injection (small)	Well-tolerated by pig, no power injector deviation
3	50	5	999	Injection (small)	Flow rate reduced by power injector due to pressure limit reached
Pig #2					
Injection Attempt	Volume (mL)	Flow rate (mL/sec)	Pressure (psi)	Port	Notes
1	45	5	1200	Injection (small)	Circuit burst where line connected to the power injector and to the port. No evidence of liver parenchymal damage
2	50	3	999	Injection (small)	Well-tolerated by pig, no power injector deviation
3	37	4	999	Injection (small)	Well-tolerated by pig, no power injector deviation
Pig #3					
Injection Attempt	Volume (mL)	Flow rate (mL/sec)	Pressure (psi)	Port	Notes
1	30	2	999	Injection (small)	Balloon slipped, rapidly dropping pressure reading
2	30	2	999	Injection (small)	Well-tolerated by pig, no power injector deviation
3	60	3	999	Injection (small)	Flow rate reduced by power injector due to pressure limit reached
4	140	1	999	Injection (small)	Well-tolerated by pig, no power injector deviation
5	80	4	999	Injection (small)	Well-tolerated by pig, no power injector deviation
6	47	10	999	Guide port (big)	Well-tolerated by pig, no power injector deviation

Three pigs were subjected to repeated hydrodynamic injections during one ERCP procedure. Different volumes, flow rates, and device catheter pressures were investigated. Clinical notes were also taken during the procedure, where any variations were reported, particularly reduction in flow rates by the power injector due to pressure limits being reached.

C57BL6 mice (4 mice) were a gift of Svetlana Lutsenko of Johns Hopkins, originally sourced from Jackson Labs. Mice were housed with littermates with water and food ad libitum, and cotton enrichment in the cage. For HTVI, C57BL/6 mice weighing between 20 and 25 grams were selected, and 2.2 mL normal saline (8–10% body weight) was subsequently injected into the lateral tail vein of mice within 4–7 seconds. At the completion of the study, mice were euthanized using carbon dioxide. Mice were harvested within 15 minutes post-injection for tissue analysis.

### Tissue analysis

A subset of animals (pigs and mice) was euthanized, underwent necropsy and were harvested for organs within 15 minutes of hydrodynamic injection. Another cohort of pigs was similarly euthanized and livers harvested on Day 1 post-injection (n = 2) or on Day 14 post-injection (n = 3), respectively, to monitor long-term effects of hydrodynamic injection. Gross inspection of the liver and abdomen was performed for each dissection. Pig livers were sampled at sites proximal and distal to the injection point in the CHD. During the dissection, the integrity of the CHD and right and left hepatic ducts in the pig liver were verified. Tissue from pig and mouse liver were fixed in 10% formaldehyde and underwent Hematoxylin & Eosin (H&E) staining.

### Blood analysis

Blood samples were collected before and after the procedure by a certified veterinary technician, the post-procedure blood draw was conducted within 15 minutes after the hydrodynamic injection. Additional blood samples were also collected before the euthanasia of pigs on Day 1 and Day 14. Blood draw was performed via the internal jugular vein of the pigs for later chemistry analysis. Liver function panel and routine serum chemistries were performed on a DiaSys Respons®910 chemistry analyzer. Samples were excluded if the chemistry analyzer showed gross hemolysis, due to its significant impact on the aspartate aminotransferase (AST), bilirubin and lactate dehydrogenase (LDH) levels. For plasmid DNA detection, DNA was isolated from serum using the QIAgen DNeasy Blood & Tissue kit, and then subjected to PCR (DreamTaq, ThermoFisher).

### Statistical analysis

GraphPad Prism 7 software (GraphPad Software) was used to perform statistical analysis and generate graphs. Unpaired, parametric, two-tailed t-tests were used to test mean differences. Significance level used was P<0.05.

## Results

### Interrogating maximum volumes and flow rates during ERCP injection

Our previous efforts defined 30 mL and 2 mL/sec as the maximally tolerated injection parameters during ERCP-mediated hydrodynamic injection [24]. At higher volumes or flow rates, the CHD upstream to the balloon ruptured, likely due to stress on the bile duct wall. In order to achieve higher flow rates and solve this issue, we adjusted our procedure for the current studies by placing the balloon immediately inferior to the liver hilum, such that the catheter tip would lie within the liver parenchyma. The pressure on the walls of bile duct walls would thus be reinforced by the liver parenchyma surrounding it, thereby preventing rupture. For our experiments, we initially used the injection port instead of the guidewire port, since the pressure catheter required the wider diameter of the guidewire port.

**Fig 1:**
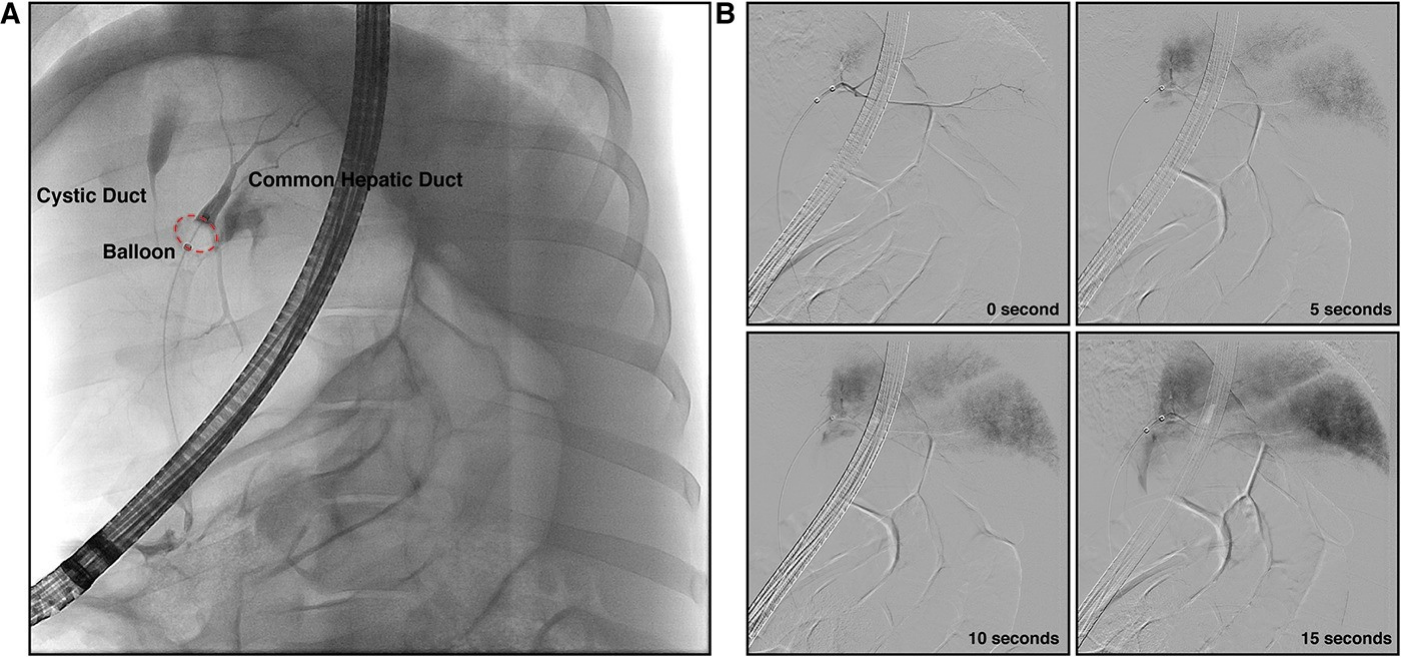
Fluoroscopy monitors the success of biliary hydrodynamic injection. (A) The anatomy of the biliary tract and the successful seal of the balloon to prevent retrograde flow of contrast was confirmed. With placement of catheter inside the CHD and balloon inflated, bifurcation of the CHD into the right and left branches is observed. (B) Hydrodynamic delivery to all lobes was confirmed by measuring real-time fluoroscopy of the injection. Example fluoroscopic images of one hydrodynamic injection are provided, showing a time course of images during injection (30 mL in 2 mL/sec). At the completion of injection (15 seconds), all liver lobes contain detectable contrast.

**Fig 2:**
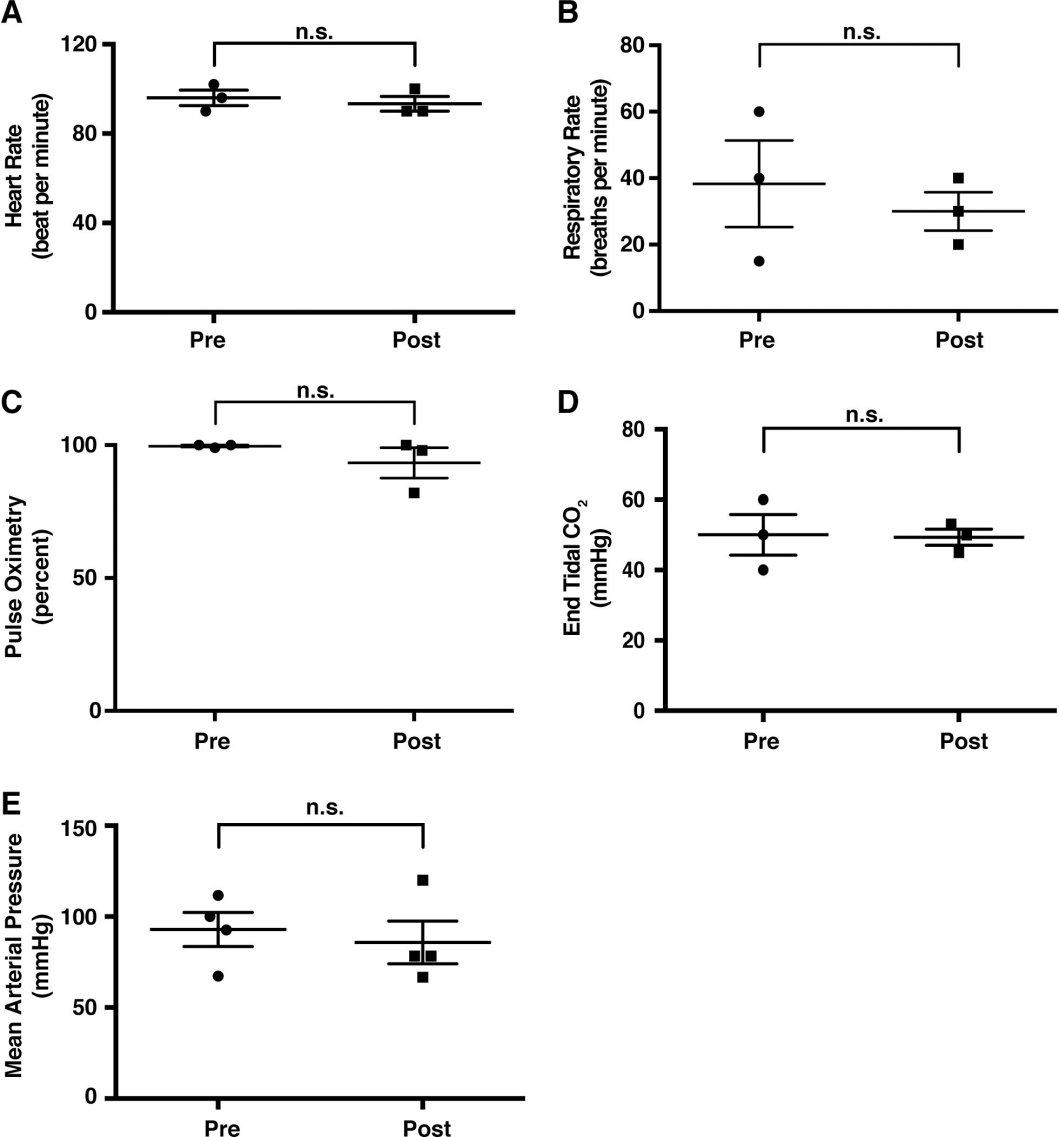
Vital signs monitored during biliary hydrodynamic injection demonstrate no significant changes. All pigs had vital signs monitored under anesthesia during ERCP procedure. Values pre- and post-procedure are provided, representing possible physiologic perturbations from hydrodynamic injection. Heart rate (A), respiratory rate (B), pulse oximetry (C), end tidal CO_2_ (D), and mean arterial pressure (E) all demonstrated no significant (n.s.) changes from pre- to post-procedure (unpaired two-tailed, parametric t-test used, P<0.05).

As in clinical practice, we first confirmed that the entire biliary tree could be visualized prior to injection (**Fig 1A**). Employing contrast solution during hydrodynamic injection, we monitored the progress of the injection during the entirety of the hydrodynamic procedure, finding the efficient flow of contrast solution into all lobes of the liver (**Fig 1B**). With the method validated, we next proceeded to test different injection parameters for their tolerability by pigs (**Table 1**). We repeated multiple hydrodynamic injections within the same pig during one operation to conserve resources and also assess toleration to multiple injections. As judged by the vital signs during procedures (heart rate, respiratory rate, pulse oximetry, mean arterial pressure, and end-tidal CO_2_) monitored before and after the injection, pigs could tolerate all the injections with no abnormalities (**Fig 2**). Representative real-time vital signs during hydrodynamic injection, including temperature, electrocardiogram and heart rate, are provided in **S1 Fig**.

As an initial test, we repeated our published parameters (30 mL at 2 mL/sec), which were well-tolerated as expected. Increasing flow rate to 4 mL/sec at the 30 mL was tolerated with no issues, but a higher volume (50 mL) and flow rate (5 mL/sec) next tested in the same pig triggered a flow rate reduction in the power injector near the end of the injection to avoid exceeding the circuit pressure limit (999 psi). Increasing the pressure limit to 1200 psi in the next pig to avoid the flow rate reduction led to the circuit tubing bursting towards the end of the injection, indicating physical limitations to the tubing and catheter materials.

Given that the smaller diameter injection port appeared to have an upper limit between 4–5 mL/sec flow rate, we switched to the guidewire channel (due to its large caliber lumen) to test tolerability to increased flow rates. We injected 47 mL at 10 mL/sec in pig #3, which tolerated this injection well with no acute changes in vital signs. The cholangiogram post-injected did not illustrate extravasation of contrast confirming the ductal anatomy remained intact.

Volume limits during biliary injection were also tested. A higher volume with a lower flow rate was tested (50 mL at 3 mL/sec) and triggered no flow rate reduction. A slightly higher volume (60 mL) at same flow rate did result in flow rate reduction during the last third of the injection. This indicated that the longer volume time adds additional wall stress to the catheter. However, when 80 mL volume was injected at 4 mL/sec flow rate in an attempt to overwhelm the biliary anatomy, no flow rate reduction occurred. The reasons for this discrepancy are unclear and could be related to physiological changes in biliary-sinusoid communication with recurrent injections. Seeing that increased volume at high flow rates may stress the system, we also asked if a larger volume at a low flow rate would similarly stress the injection system or the pig’s vital signs. A 140 mL of volume, near the volume limit of the power injector, at 1 mL/sec was well tolerated with no change in vital signs, and the power injector had no issues.

### Pressure monitoring during hydrodynamic ERCP injection

**Fig 3:**
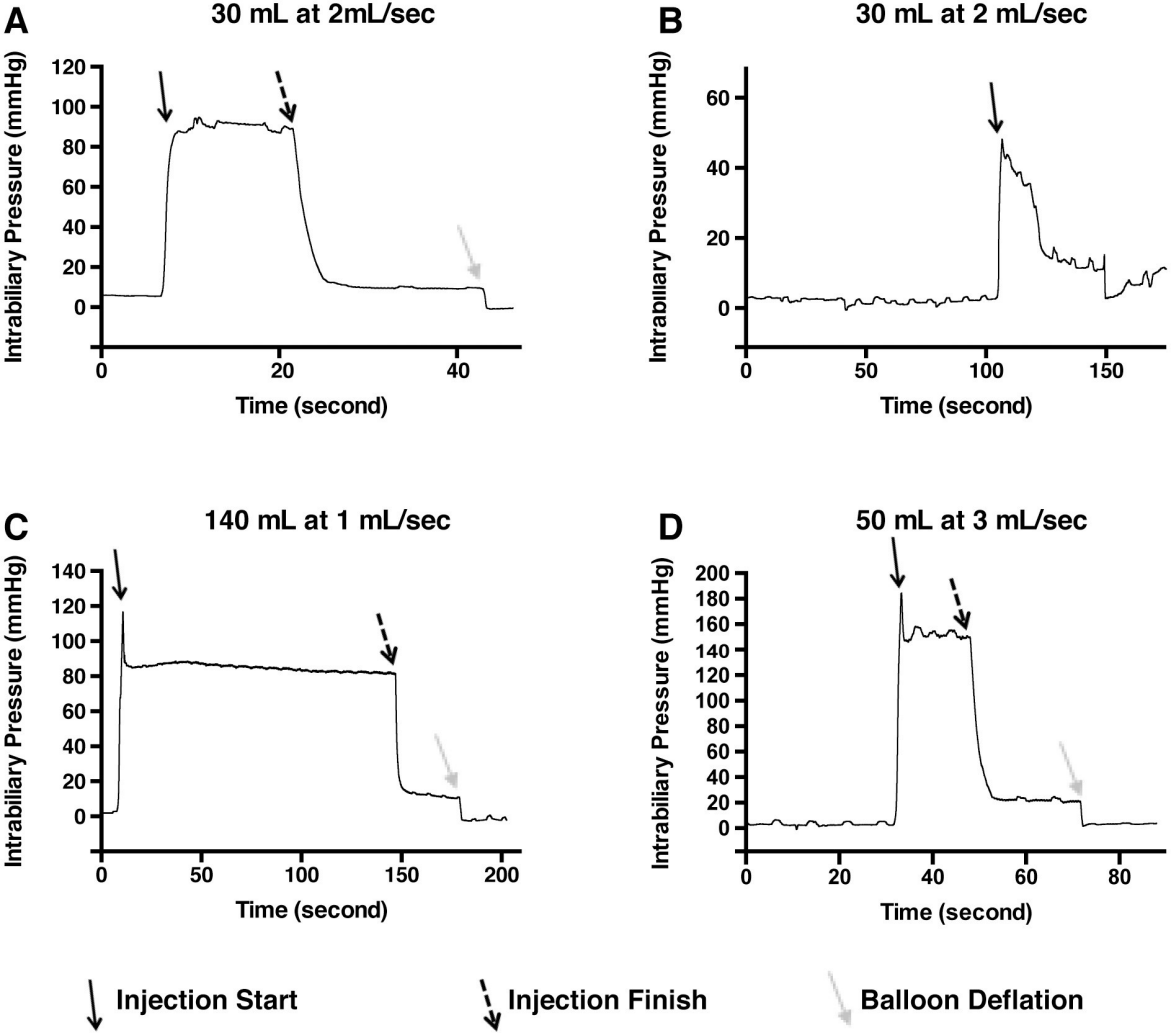
Intrabiliary pressure monitored during hydrodynamic injection elucidates differences among the injection parameters. Baseline pressure within the biliary system is minimal before injection in all conditions (A-D). Shortly before injection, the balloon is inflated creating a seal which appears to have minimal effect on the measured pressure. Upon initiation of injection (solid black arrow, parameters provided above graphs), pressure increases to a short peak, before equilibration during flow at a slightly lower pressure. Cessation of injection (dashed black arrows) yields a sharp decrease in pressure. The deflation of balloon (solid grey arrows) drops pressure further, suggesting a measure of baseline hydrostatic pressure remains in the system after the injection is completed. Higher pressure was achieved at the highest flow rate (D).

Pressure achieved during hydrodynamic injections was also evaluated, given its importance to the efficacy of hydrodynamic delivery in rodent models [8]. A pressure sensing probe was inserted through the guidewire lumen and successfully positioned 1 cm upstream of the catheter tip. Pressure readings for the injection of 30 mL at 2 mL/sec demonstrated a plateau pressure of 80 mmHg during injection, that promptly dropped the moment the injection ended (**Fig 3A**). A small level of pressure was released when the balloon was deflated, probably representing pressure generated by balloon restriction of biliary flow, although the exact value was variable between the different experiments (4.23 mmHg to 18.92 mmHg). A peak pressure point at the initial power injector was also noted in two of the conditions (114.76 mmHg in **Fig 3C** and 181.36 mmHg in **Fig 3D**) before slightly falling into a plateau phase. This peak pressure point likely represents the pressure in the biliary system immediately before fluid begins escaping into the vascular system, and the plateau phase may represent a steady-state pressure of fluid entry and exit into the vascular system.

The pressure curve was also able to detect the balloon accidentally slipping backward, releasing fluid into the gallbladder (**Fig 3B**) as confirmed using fluoroscopy. Pressure monitoring may thus be useful to routinely confirm successful injection. The flow rate to pressure relationship appears to be non-linear, since a 1 mL/sec injection and 2 mL/sec injection both similar pressure, 82.12 mmHg and 89.12 mmHg, respectively, during injection, while the 3 mL/sec injection yielded 148.58 mmHg (**Fig 3C and 3D**). We were unable to perform pressure measurements during the other pig injections, but our results suggest the potential for even higher pressures to be achieved at higher flow rates. A summary of the achieved pressures achieved during hydrodynamic injection is provided in **S1 Table**.

### Organ damage and tissue analysis

Acute pathogenic changes occurring in pigs immediately post-procedure after repeated hydrodynamic injections were next examined. Pigs were sacrificed within 15 minutes of the last injection, and all showed grossly normal anatomy upon examination without swelling, bruising, or rupture (**S2 Fig**). The CHD was probed with an instrument in Pig #1, confirming intact anatomic status with no gross lesions (**S2C Fig**). The diaphragmatic and visceral surfaces were intact in the pig (**S2A and S2B Fig**). Looking at blood sampled pre- and post-injection, aspartate aminotransferase (AST) showed a notable increase from 19 U/L to 137 U/L in pig #2 and 59 U/L to 252 U/L in pig #3 (**Table 2**). All other measurements, including alanine aminotransferase (ALT) remained within normal limits.

**Table 2:** Serum chemistry before and after repeated biliary hydrodynamic injections in pig liver was evaluated.

	Pig #1		Pig #2		Pig #3		Normal Reference
	Pre	post	pre	post	pre	post	
AST (units/L)	44	48	19	137	59	252	32–84
ALT (units/L)	57	56	51	49	88	90	31–58
Amylase (relative units)	1752	1584	1853	1464	758	705	
Albumin (g/dL)	3.3	2.7	3.5	3.3	3.5	3.2	1.9–3.9
Total bilirubin (mg/dL)	0.3	0.5	0.2	0.6	0.3	0.3	0–10
Direct bilirubin (mg/dL)	0.2	0.5	0.2	0.5	0.2	0.3	0–0.3
Creatinine (mg/dL)	1.9	1.6	1.9	1.8	1.7	1.6	1.0–2.7

AST, aspartate aminotransferase; ALT, alanine aminotransferase.

References: [36]

A panel of chemistry tests measuring liver function was performed on pre- and post-treatment samples. AST showed an acute rise in Pig #2 and Pig #3, while Pig #1 remained within normal limits. Total and direct bilirubin showed increases in Pig #1 and Pig #2 post-injection, although the increase remained within normal limits. All other values showed no significant changes.

**Fig 4:**
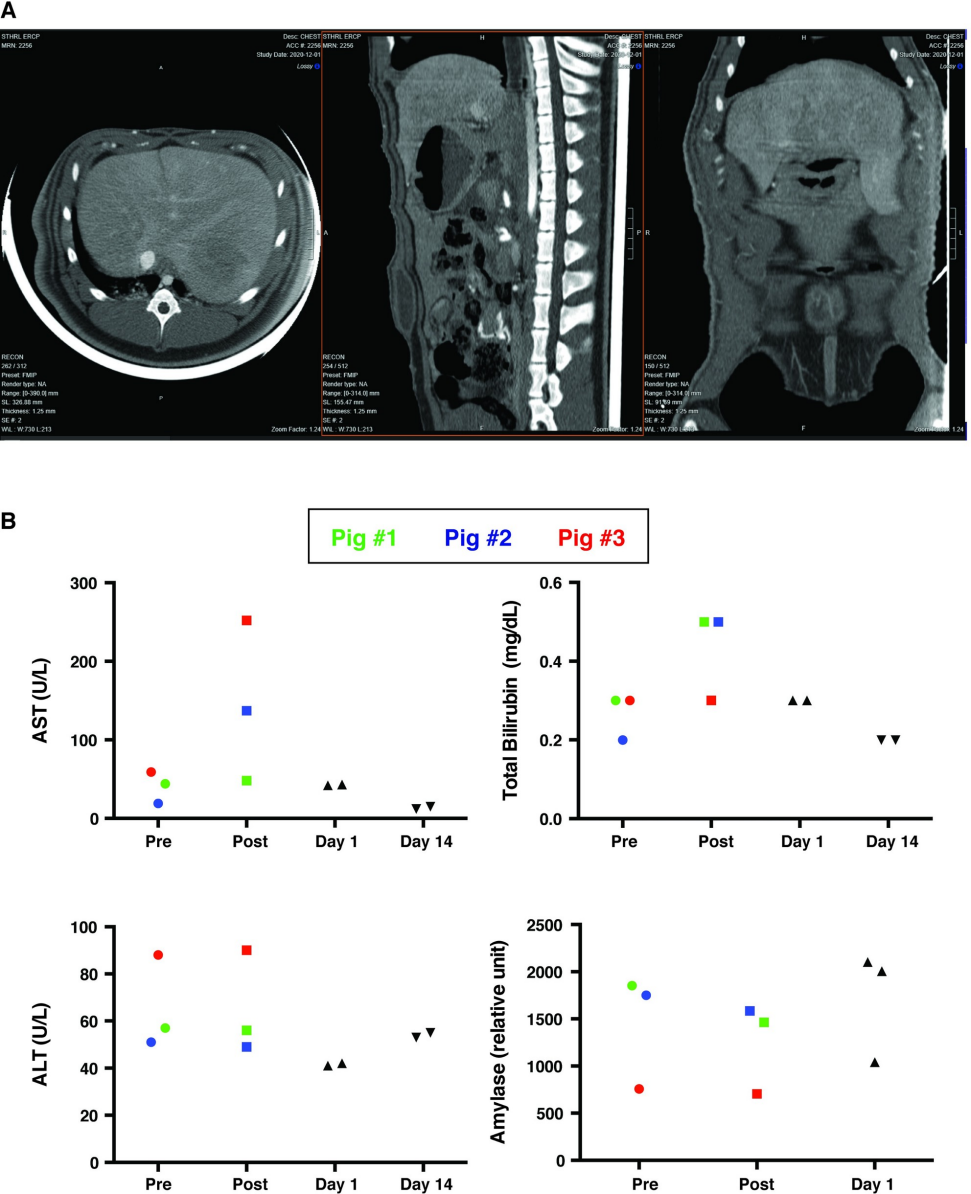
Imaging and biochemical analysis support the safety profile of biliary hydrodynamic injection. (A) Abdominal CT with contrast was performed on Day 1 post-procedure. The axial, sagittal and coronal images did not demonstrate any evidence of intra- or extrahepatic biliary dilation, hepatic infarction/necrosis, abnormal gallbladder dilation, or gallbladder inflammation. The systemic and portal venous systems were patent. (B) Transient, acute elevation in AST and total bilirubin in the three injected was noted, which were not observed in five other pigs with resolution of any biochemical abnormalities by day 1 post-injection. Pig #1 was color-coded as green dots, Pig #2 was color-coded as blue dots, Pig #3, which received multiply injections with the largest volume and highest injection speed, was color-coded as red dots.

Beyond monitoring biochemical markers for injury, abdominal imaging was performed to evaluate injury from hydrodynamic injections. Abdominal CT with contrast was performed on Day 1 post-injection at parameters of 4 mL/sec and 40 mL of volume. Axial, sagittal and coronal images did not demonstrate any evidence of intra- or extrahepatic biliary dilation, and liver did not show any sign of injury with lack of infarction or necrosis (**Fig 4A**).

Short and long-term toxicity from injection was also characterized 15 minutes after injection, as well as on Day 1 and Day 14 post-injection (**Fig 4B**). While there was a transient elevation in AST and total bilirubin at 15 minutes post-injection, these values returned to normal range on Day 1 post-injection and remained within normal range 14 days post-injection (**Fig 4B**). ALT and amylase did not show any obvious change after hydrodynamic injection at any time point.

To investigate where this excess fluid went during the biliary injection, plasmid DNA was dissolved into the solution and injected into one of the pigs. PCR targeted to an internal sequence on the plasmid DNA was performed on DNA isolated from serum collected at all time points. Plasmid DNA was detected in serum 15 minute post-injection illustrating passage from bile into vascular circulation and was no longer detectable in serum on day 1 post-injection (**S3 Fig**).

**Fig 5:**
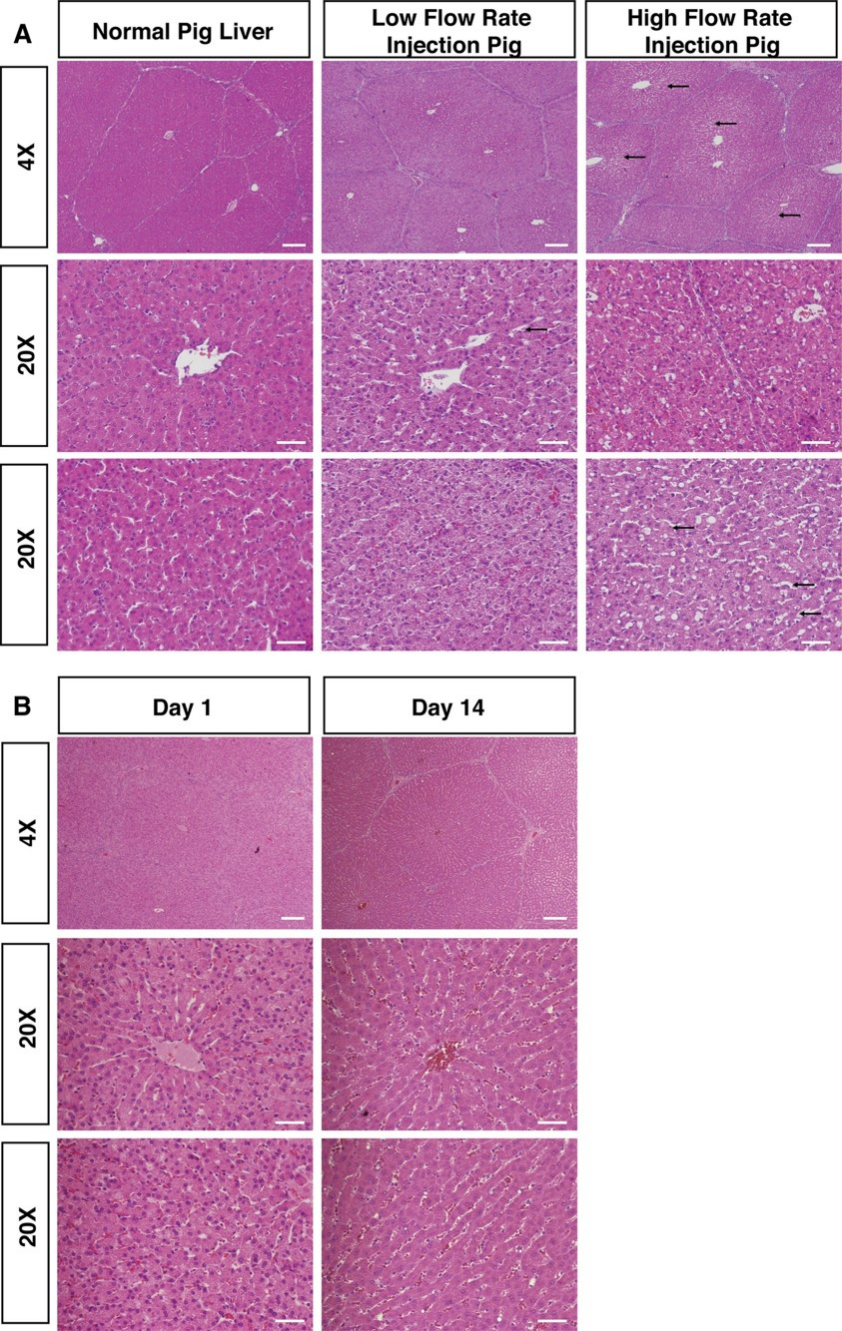
Histology of pig liver post-repeated hydrodynamic biliary injection shows evidence of hydrodynamic effects. (A) H&E stains from low flow rate pig injections (flow rates: <5 mL/sec; Pig #1 depicted) or high flow rate pig injections (flow rate: 10 mL/sec; Pig #3 depicted) are illustrated, along with H&E from a normal, non-injected pig liver for comparison. Histology taken from livers 15 minutes post-injection showed significant dilation of hepatic sinusoids (black arrows) and formation of intracellular vesicles, but otherwise no areas of focal necrosis. (B) Histology of pig liver on Day 1 and Day 14 post-procedure showed normal liver histology. H&E staining on pigs euthanized on Day 1 and Day 14 post-procedure are illustrated with no obvious sinusoid spaces dilation observed. Scattered fluid-filled vesicles were observed more rarely on Day 1 post-procedure. No fluid-filled vesicles were noted on Day 14. Scale bar: 200 μm in 4X images; 50 μm in 20X images.

Liver histology in pig #3 acutely injected at the higher flow rate demonstrated larger dilation of sinusoid spaces within hepatic lobules compared to both pigs injected at lower flow rates (**Fig 5A**), consistent with fluid rapidly exiting the biliary canaliculi and expanding sinusoidal spaces [21]. Central veins appeared to be the same size between injected and non-injected animals, while the hepatocyte cytoplasm appeared to be more dilute in the injected pigs than an un-injected pig (**Fig 5A**), resulting from intracellular entry of fluid [14]. Pig #3 exhibited numerous large, intracellular fluid-filled vesicles scattered throughout the hepatocyte cytoplasm, which were not observed in pig #1 and pig #2 (**Fig 5A**). These effects were observed in proximal and distal segments to injection of all five lobes of pig #3, suggesting pressure was able to be distributed evenly throughout the entire organ (**S4 Fig**).

To evaluate the long-term impact of biliary hydrodynamic injection, histological analysis of pigs euthanized on Day 1 and Day 14 post-injection was performed. There was no obvious dilation of sinusoid spaces. Scattered fluid-filled vesicles were still able to be noted on Day 1 post-procedure but were much less compared to 15 minutes post-injection. No fluid-filled vesicles were noted on Day 14 post-injection (**Fig 5B**). Looking at biliary injury from hydrodynamic injection, the morphology of large, medium and small bile ducts showed no apparent rupture at the highest flow rates tested, looking histologically similar to un-injected, control pig liver (**S5A Fig**). Similarly, the epithelium lining of the intrahepatic and extrahepatic bile duct was intact, while peribiliary glands kept integrity (**S5B Fig**).

### Comparison to murine hydrodynamic tail vein injection

**Fig 6:**
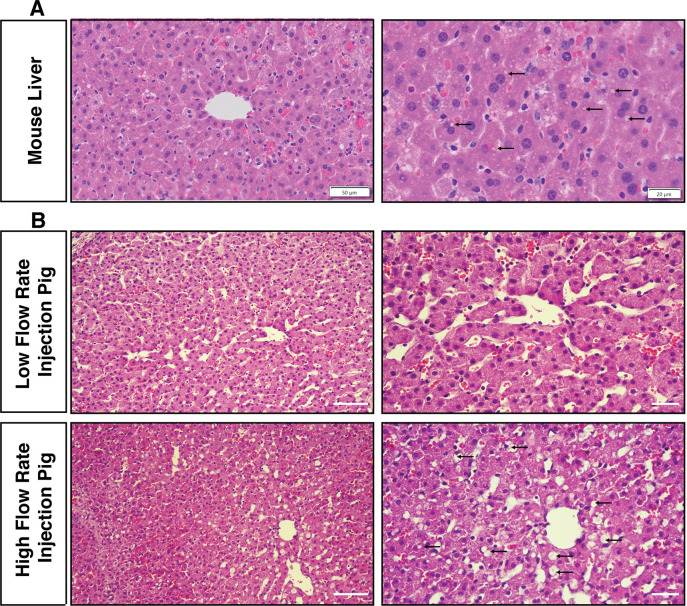
Hydrodynamic biliary injection induces acute tissue structural changes in pigs that is similar to mouse hydrodynamic tail vein injection. (A) A mouse was injected with 10% body fluid volume over 4–7 seconds through the tail vein and sacrificed 15 minutes later for comparison to the biliary hydrodynamic injection in pigs. Small fluid-filled cytoplasm vesicles (black arrows), scattered hepatocytes with dilute cytoplasm, and occasional hepatocytes with engulfed red blood cells are observed in the mouse liver. Scale bar: left, 50 μm; right, 20 μm. (B) Pig #3, which is a high flow rate injection pig, has largely similar changes, with larger fluid-filled cytoplasmic vesicles and more frequent hepatocytes with dilute cytoplasm. Scale bar: left, 50 μm; right, 2.0 μm.

We finally sought to compare the histopathology of the mouse liver shortly after HTVI with the histopathology of pig liver after biliary hydrodynamic injection. Scattered hepatocytes contained dilute cytoplasm in mouse liver, along with occasional hepatocytes containing red blood cells, the latter reflective of the vascular route of the procedure (**Fig 6**). Numerous fluid-filled vesicles were seen within murine hepatocytes, although generally smaller than the vesicles seen in pig #3. The combination of histological changes most resembles the high-pressure injections in pig #3, suggesting these high pressure/flow rates generated could mimicked HTVI in mice effectively.

## Discussion

In this study, we performed a systematic characterization of hydrodynamic injection parameters during ERCP as method for liver directed gene therapy. We characterized novel injection parameters *in vivo* in pigs, and found that the positioning of the balloon within the intrahepatic CHD allowed higher volumes and higher flow rates during injection than in our previous testing [24]. Injected fluid escapes into the vascular system, with pressure measured correlating with increases in flow rate (up to 150 mm Hg measured). Flow rate also correlated with histological findings in pig liver, with similar fluid-filled vesicles to mouse hydrodynamic injection. Additionally, we identified potential areas for improvement in current clinical equipment to optimize the procedure going forward.

Our studies on injection volume discovered no clear upper limit during biliary injection. Volume only seemed to be important with regards to prolonging time the catheter walls were subjected to stress under high flow rates, which caused pressure thresholds to be reached. As evidenced by the detection of plasmid DNA in the serum, the injected fluid exits from bile canaliculi and through junctions between hepatocytes and into the space of Disse and sinusoids [21]. Interestingly, likely due to the relatively small volumes injected, contrast was not seen on fluoroscopy in the hepatic veins or inferior vena cava, although radiocontrast can be detected in peripheral circulation post-ERCP [26]. Permeability of canalicular tight junctions in the setting of DNA transfer has been observed in a rat model previously [21], and we demonstrate here that is occurs in pigs as well, and thus likely would happen in human patients.

Our studies on pressure during injection demonstrated that flow rate appeared to be the key determinant, closely correlating with the injection initiation and cessation. A flow rate of 5 mL/sec appeared to be the highest achievable via the injection channel of the catheter before approaching circuit pressure limit and triggering flow rate reduction by the power injector, while the larger guidewire channel tolerated at least 10 mL/sec flow rate. Compared to vascular hydrodynamic pig studies, the plateau pressure of 148 mmHg during 3mL/sec injection is similar to pressure achieved in other studies through vascular routes that demonstrated gene delivery (**Table 3**) [13, 17, 27–32]. These other studies often employed much higher flow rates to achieve these pressures, suggesting that compliance and greater volume (~600 mL) [33] of the venous system handicap vascular approaches. By contrast, the relatively small diameter and/or volume of the biliary system (estimated 29 mL in humans [34]) should serve to rapidly increase the pressure even at low flow rates, as compared to higher flow rates required to achieve similar pressure from vascular approaches. We note the potential even higher pressures being achieved, given that we did not record measurements for 4 mL/sec, 5 mL/sec, and 10 mL/sec, respectively.

**Table 3:** Comparison of intravascular pressures achieved in previous hydrodynamic liver gene therapy studies.

Year	PMID	Species	Description of Procedure and Pressure Achieved
2005	15729372	Mouse	Achieved 20–30 mmHg pressure in portal vein and IVC after hydrodynamic tail vein injection
2006	16871229	Pig	150 mL injected at 3 mL/sec (achieving 44 mmHg portal vein pressure) and at 5 mL/sec (58 mmHg achieved)
2008	18004400	Pig	360–400 mL injected at 100mL/sec achieving 101–126 mmHg; clamped IVC for delivery
2009	19156134	Pig	600 mL injected at 40 mL/sec, achieved of 75 mmHg in hepatic vein; pressure up to 100–125 mmHg with IVC occlusion
2011	21091276	Pig	200mL injected at 50 mL/sec in isolated lobe, peaking perfusion pressure 103.9 and 226.7 mmHg in two pigs
2013	24129227	Pig	600 mL injected at 40 mL/sec, catheter advanced into specific liver lobes through hepatic vein. Proximal site achieved 100 mmHg, while distal site was 200 mmHg.
2015	26398117	Pig	30 mL injected at 20 mL/sec into 4 week old pigs at weaning; portal vein pressure 93 mmHg achieved
2017	28447859	Dog	200 mL injected at 20mL/sec yielding peak intravascular pressure between 85–140 mmHg

Studies exploring hydrodynamic gene delivery in mouse, pigs, and dogs are listed, along with the reported intravascular pressure achieved in them. These comparisons show that the biliary hydrodynamic injection strategy compares favorably to these approaches (~150 mmHg at 3mL/sec) with significantly less volume and flow rate utilized.

To summarize the mechanism of these findings, biliary hydrodynamic injection rapidly increases pressure in the biliary system (peak pressure) before reaching a plateau of steady-state pressure of infusion and escape into the vascular space, explaining wide toleration of volume in the procedure. Importantly, no significant changes in vital signs were noted before and after procedures, regardless of volume or flow rate. This differs from intravascular hydrodynamic injections in pig liver, wherein modulation of heart rate, blood pressure, and respiratory rate occur during balloon occlusion and opening [17]. Long-term, the human application could optimally use as little volume as possible while balancing transfection efficiency, in order to avoid any effect on rapid increases in intravascular volume.

Another encouraging discovery was the histological findings of large, fluid-filled vesicles in the cytoplasm of pig hepatocytes, which were also observed in the mouse hepatocytes injected by hydrodynamic tail vein injection (**Fig 6**), as seen in other studies [13]. Fluid-filled vesicles are speculated to be directly from pinched off cytoplasmic membrane, resembling macropinocytosis vesicles [14], and may help deliver DNA during hydrodynamic injection, as an alternative to direct transfer through cytoplasmic membrane and nuclear pores [8]. This study is the second time that generation of fluid vesicles have been achieved in large animals after hydrodynamic injection [35]. Given that the lower flow rate parameters failed to induce these vesicles, we speculate that these higher flow rates could mediate higher gene delivery efficiencies. Importantly, despite the large volume and flow rate employed, bile ducts themselves were observed to be intact and un-injured after hydrodynamic injection.

This study also defines important parameters concerning liver damage induced by different injection parameters. A mild increase in AST (252 U/L) occurred in pig #3 injected at the highest flow rate, which resolved in other pigs by day 1 and day 14 post-injection. At lower flow rates in pig #1, no elevation in liver enzymes occurred. Together, these findings suggest a wide range of tolerability to differ injection parameters. Another important finding was the influence of psi settings in the power injector; 999 psi was tolerated, while 1200 psi broke the tubing. This limitation could be resolved with catheter materials optimized for this application in the future at higher tensile strength.

Our long-term, two week data post-injection demonstrated the normalization of liver function, which was expected based on previous reports of hydrodynamic injection in mice, dogs, and pigs [9, 10]. That said, additional studies should be conducted at even higher flow rates to confirm normalization of liver histology. Moreover, while validating the ability of pigs to tolerate repeated injection is enticing for strategies to increase transfection efficiency, there is a risk that the prior injections may have altered the liver tissue. Thus, studies should repeat high volume, flow rate parameters on injection naive pigs to ensure similar results.

In conclusion, we have identified new injection parameters, safety data and constraints of hydrodynamic injection via ERCP into pigs, while replicating aspects of the technique’s mechanism from mice to pigs. We also confirmed the permeability of the biliary system in pigs for the first time. Given that humans and pigs have similar liver size and anatomy and that we employed clinical instruments in our procedures, we believe these parameters are applicable toward improving gene delivery methods in human patients. Beyond gene therapy, our findings may be applicable to development of new applications of ERCP, where large injected volumes or flow rates could be used.

## Supporting information

S1 Fig Representative real-time vital signs during hydrodynamic injection, including temperature, electrocardiogram and heart rate. Continuous measurements were taken throughout one of the biliary hydrodynamic injection procedures in pigs. There was no change in temperature, heart rate or electrocardiogram at parameters of injection of 4 mL/sec over 10 seconds.
(TIF)

S2 Fig Gross examination of livers of pigs immediately post-injection series does not show any obvious abnormalities. The visceral surface (A) and diaphragmatic surface (B) of pig #1 are depicted, showing lobes with no obvious lesions. The common hepatic duct (CHD) of pig #1 where the catheter was placed for injection was further probed (C) demonstrating no wall lesions or tears. The visceral surface of pig #2 (D) and pig #3 (E) are also shown after tissue harvest post-injection, also demonstrating no obvious gross abnormalities.
(TIF)

S3 Fig PCR for plasmid DNA in serum samples illustrates the permeability of the biliary system. To evaluate the escape of fluid from the biliary system during injection, plasmid DNA (pCLucf) was diluted into the injection solution. PCR primers were designed to target the GFP sequence in the pCLucf plasmid. PCR was performed on the serum samples obtained pre-injection, 15 min post-injection and on day 1 post-injection. The DNA molecule was detected in the 15 min post-injection sample, which was no longer detectable on day 1 post-injection. The PCR band size over the GFP gene: ~300bp. Negative control used molecular water as template DNA. Bright band in the ladder represents 500 bp, and each ladder band below is at intervals of 100 bp.
(TIF)

S4 Fig Vesicle formation is observed in the proximal and distal portions of liver lobes at high flow rates. H&E stain from high flow rate injection pig #3 (10 mL/sec) is illustrated across the five liver lobes and sampling proximal and distal to the common hepatic duct injection site. LLL, left lateral lobe; LML, left medial lobe; RML, right medial lobe; RLL, right lateral lobe. Scale bar: 100 μm.
(TIF)

S5 Fig Bile ducts do not show signs of injury or rupture from biliary hydrodynamic injection. Liver histology of the pig injected at the highest flow rates tested (10mL/sec) was assessed in a liver collected 15 minutes after injection. (A) The morphology of large, medium and small bile duct exhibits no gross differences in a pig injected at high flow rates compared to an un-injected, normal pig liver control. (B) The epithelium lining of the intrahepatic and extrahepatic bile duct was also intact in the same animal, while the peribiliary glands maintained integrity. Scale bar: 50 μm.
(TIF)

S1 Table Intrabiliary pressure values during hydrodynamic injection in pigs. Pigs were monitored during biliary hydrodynamic injection at different volumes and flow rates. Pressure values (mmHg) captured during monitoring are provided for peak pressure, steady-state pressure, and post-injection pressure before balloon deflation.
(PDF)
