# Supplementary figures and images for: Parameters of biliary hydrodynamic injection during endoscopic retrograde cholangio-pancreatography in pigs for applications in gene delivery
Source: PLoS One. 2021 Apr 28;16(4):e0249931. doi: 10.1371/journal.pone.0249931 (PMC8081268; doi:10.1371/journal.pone.0249931)

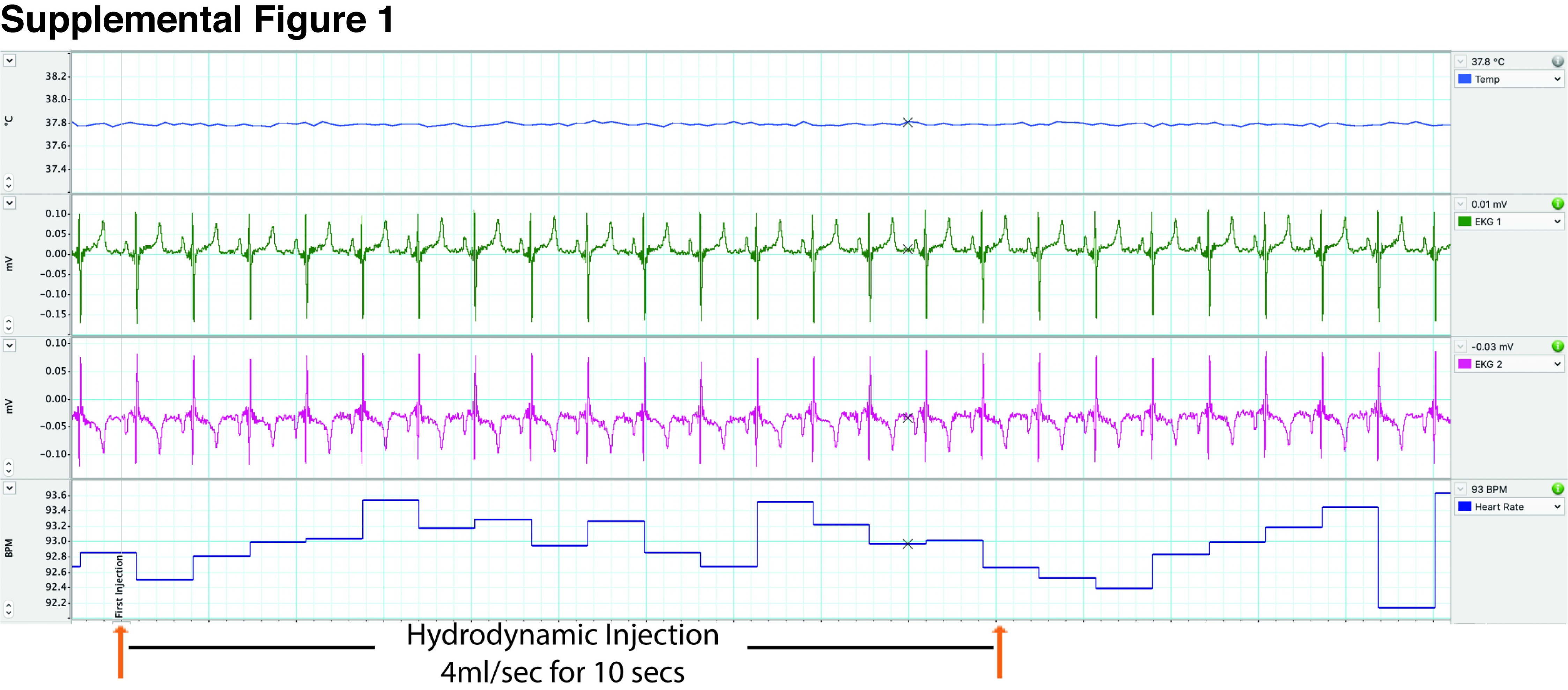

Supplement: S1 Fig — Continuous measurements were taken throughout one of the biliary hydrodynamic injection procedures in pigs. There was no change in temperature, heart rate or electrocardiogram at parameters of injection of 4 mL/sec over 10 seconds. (TIF) [file pone.0249931.s001.tif]

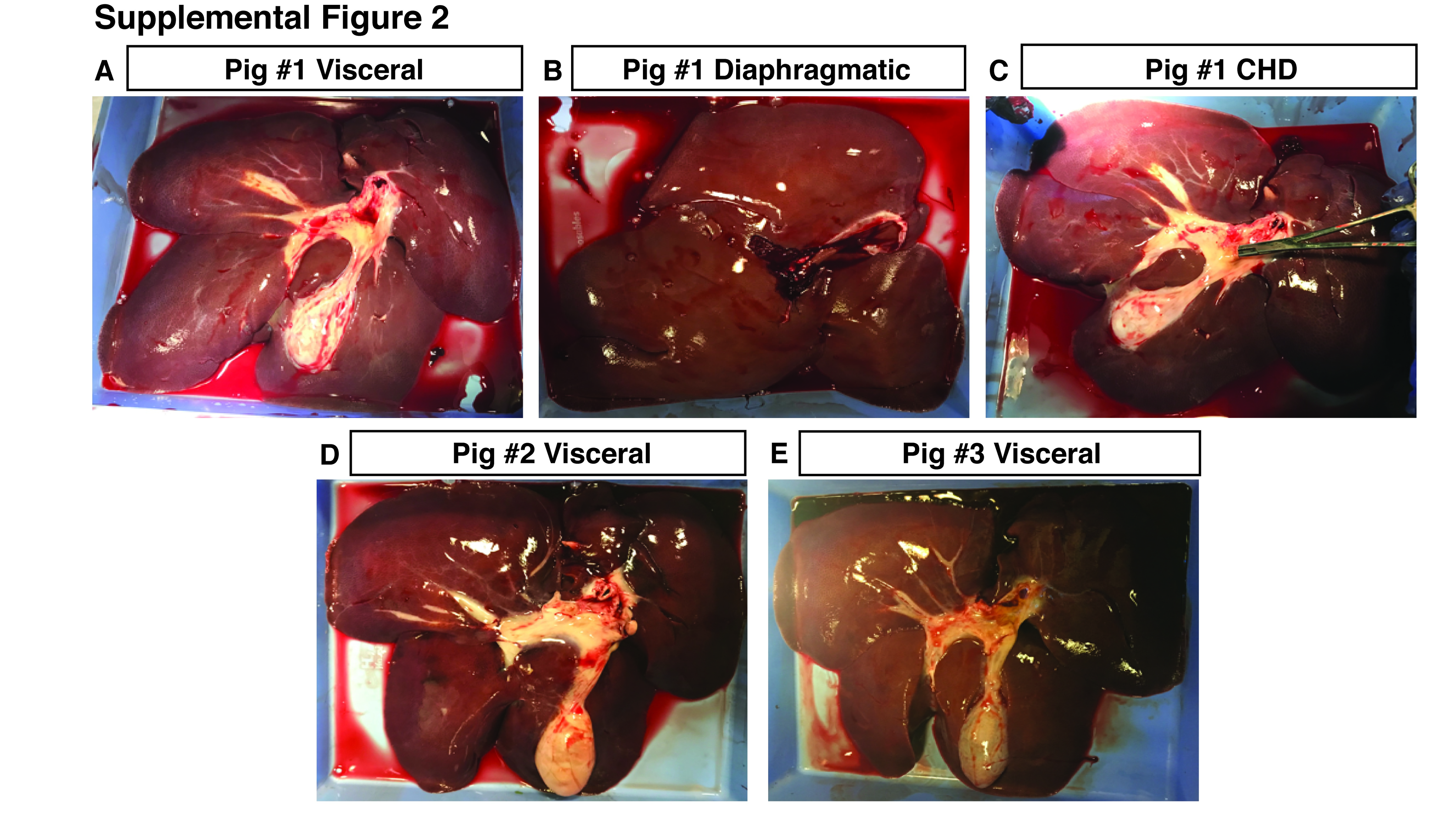

Supplement: S2 Fig — The visceral surface (A) and diaphragmatic surface (B) of pig #1 are depicted, showing lobes with no obvious lesions. The common hepatic duct (CHD) of pig #1 where the catheter was placed for injection was further probed (C) demonstrating no wall lesions or tears. The visceral surface of pig #2 (D) and pig #3 (E) are also shown after tissue harvest post-injection, also demonstrating no obvious gross abnormalities. (TIF) [file pone.0249931.s002.tif]

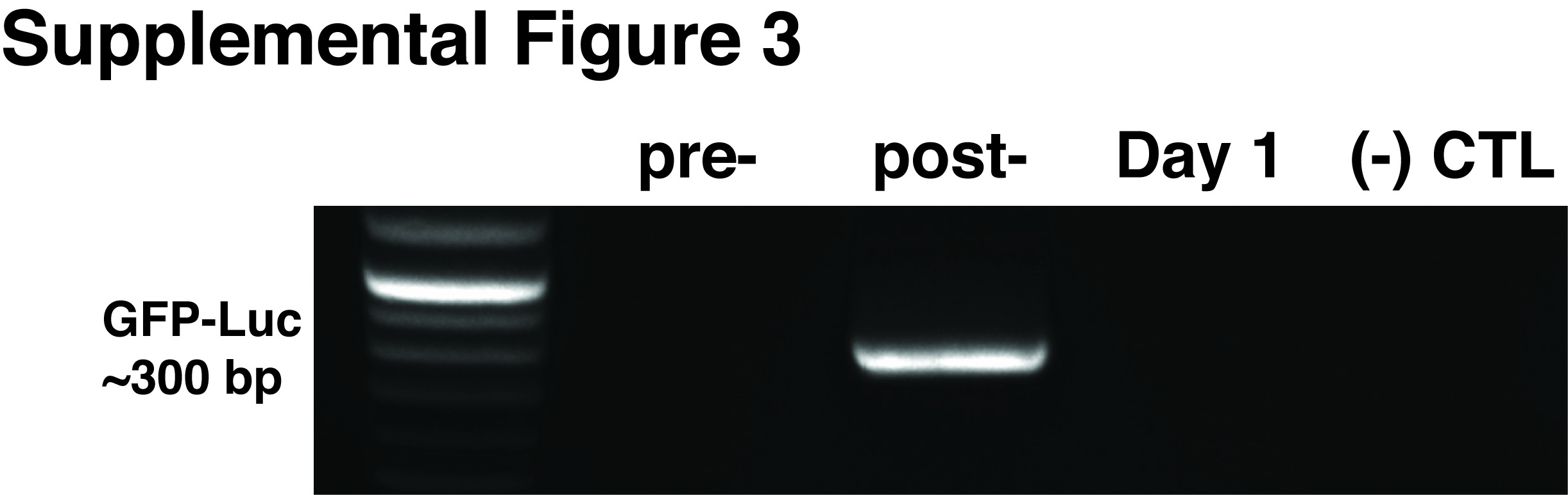

Supplement: S3 Fig — To evaluate the escape of fluid from the biliary system during injection, plasmid DNA (pCLucf) was diluted into the injection solution. PCR primers were designed to target the GFP sequence in the pCLucf plasmid. PCR was performed on the serum samples obtained pre-injection, 15 min post-injection and on day 1 post-injection. The DNA molecule was detected in the 15 min post-injection sample, which was no longer detectable on day 1 post-injection. The PCR band size over the GFP gene: ~300bp. Negative control used molecular water as template DNA. Bright band in the ladder represents 500 bp, and each ladder band below is at intervals of 100 bp. (TIF) [file pone.0249931.s003.tif]

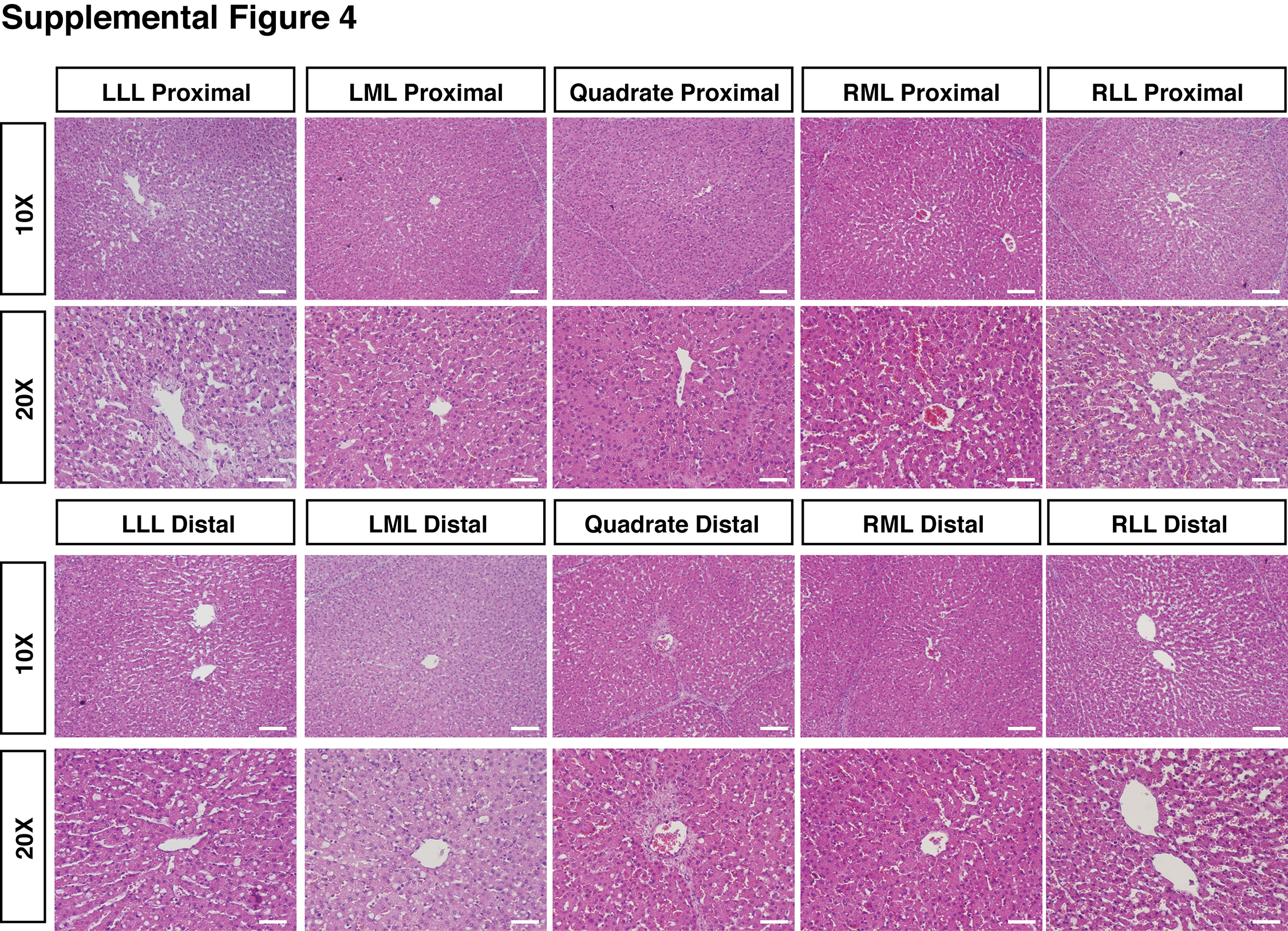

Supplement: S4 Fig — H&E stain from high flow rate injection pig #3 (10 mL/sec) is illustrated across the five liver lobes and sampling proximal and distal to the common hepatic duct injection site. LLL, left lateral lobe; LML, left medial lobe; RML, right medial lobe; RLL, right lateral lobe. Scale bar: 100 μm. (TIF) [file pone.0249931.s004.tif]

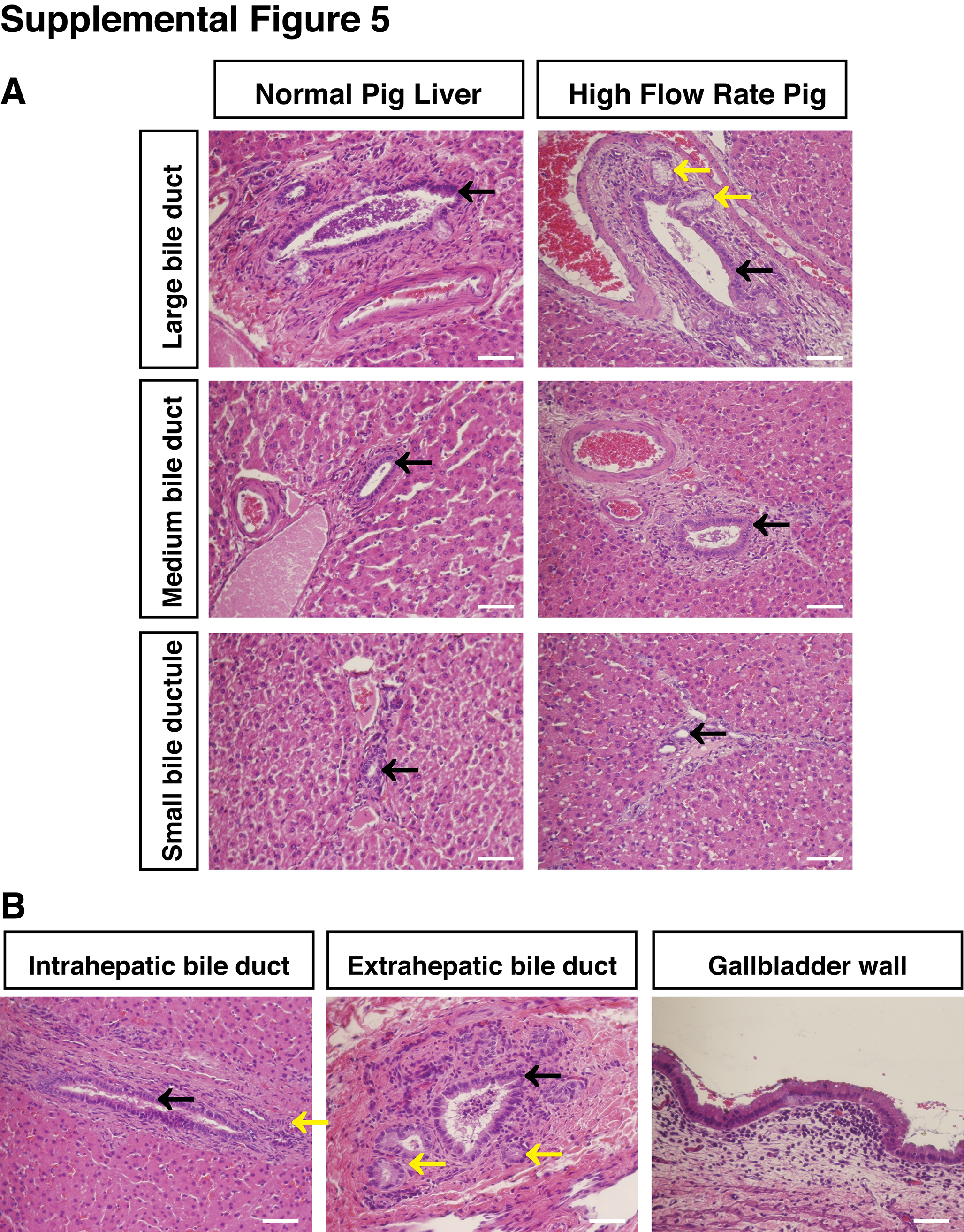

Supplement: S5 Fig — Liver histology of the pig injected at the highest flow rates tested (10mL/sec) was assessed in a liver collected 15 minutes after injection. (A) The morphology of large, medium and small bile duct exhibits no gross differences in a pig injected at high flow rates compared to an un-injected, normal pig liver control. (B) The epithelium lining of the intrahepatic and extrahepatic bile duct was also intact in the same animal, while the peribiliary glands maintained integrity. Scale bar: 50 μm. (TIF) [file pone.0249931.s005.tif]
