# Supplementary material for: Parameters of biliary hydrodynamic injection during endoscopic retrograde cholangio-pancreatography in pigs for applications in gene delivery
Source: PLoS One. 2021 Apr 28;16(4):e0249931. doi: 10.1371/journal.pone.0249931 (PMC8081268; doi:10.1371/journal.pone.0249931)
Supplement: S1 Table — Pigs were monitored during biliary hydrodynamic injection at different volumes and flow rates. Pressure values (mmHg) captured during monitoring are provided for peak pressure, steady-state pressure, and post-injection pressure before balloon deflation. (PDF) [file pone.0249931.s006.pdf]

S1 Table. Intrabiliary pressure values during hydrodynamic injection in pigs.

Trial Name	Volume (mL)	Flow rate (mL/sec)	Peak Pressure during Injection (mmHg)	Steady-State Pressure during Injection (mmHg)	Pressure before Balloon Deflation (mmHg)
Pig #2, Trial #1	50	3	181.36	148.58	18.92
Pig #3, Trial #1	30	2	46.08	36.42	10.71
Pig #3, Trial #2	30	2	89.12	85.06	4.23
Pig #3, Trial #3	140	1	114.76	82.49	9.98

Pigs were monitored during biliary hydrodynamic injection at different volumes and flow rates. Pressure values (mmHg) captured during monitoring are provided for peak pressure, steady-state pressure, and post-injection pressure before balloon deflation.
